# Selective inhibitors of nuclear export (SINE) in hematological malignancies

**DOI:** 10.1186/s40164-015-0002-5

**Published:** 2015-03-01

**Authors:** Arundhati Das, Guoqing Wei, Kaushal Parikh, Delong Liu

**Affiliations:** Department of Medicine, Westchester Medical Center, Valhalla, NY 10595 USA; Bone Marrow Transplantation Center, The First Affiliated Hospital, Zhejiang University School of Medicine, Hangzhou, China; Henan Cancer Hospital, Zhengzhou University, Zhengzhou, China

**Keywords:** Selective inhibitor of nuclear export, SINE, Slinexor, KPT-330

## Abstract

Regulated nucleo-cytoplasmic transport plays a major role in maintaining cellular homeostasis. CRM1 (chromosome region maintenance 1 or exportin 1 or XPO 1) is responsible for the nucleo-cytoplasmic transport of more than 200 proteins, including most of the tumor suppressor proteins (TSP). CRM1 is overexpressed in pancreatic cancer, osteosarcoma, glioma, cervical and hematological malignancies. This inspired the development of novel agents that selectively inhibit nuclear exportins (SINEs). In this review we focus on the significance of CRM1 in carcinogenesis and review the new development of SINE inhibitiors in hematological malignancies. Selinexor (KPT-330) as the first-in-human SINE agent represents this novel class of anti-cancer agents.

## Introduction

Regulated nucleo-cytoplasmic transport plays a major role in maintaining cellular homeostasis. The mode of transport across nuclear membrane depends primarily on the size of the molecules. Small molecules undergo passive diffusion through a protein complex called nuclear core complex (NPC). However, the transport of macromolecules (>40Kda, “cargo”), requires an association between transport receptors and the NPC [1-3].

The transport receptors belong to the karyopherin Beta (Kap) family. Nineteen of them are present in the human cells. These include importins, exportins and bidirectional Kaps. The association between the NPC, transport receptors and cargo proteins is controlled by a Ran GTPase [4].

There are 7 export proteins or exportins in the Kap family, such as CRM1, cas, exportin, etc. The CRM1 (chromosome region maintenance 1 or exportin 1 or XPO 1) is responsible for the transport of more than 200 proteins, including most of the tumor suppressor proteins (TSP). The relocation of TSPs to the cytoplasm results in their inactivation that leads to carcinogenesis [2].

CRM1 is overexpressed in pancreatic cancer, osteosarcoma, glioma, cervical and hematological malignancies. This inspired the development of novel agents that selectively inhibit nuclear exportins (SINEs) [4,5]. In this review we focus on the significance of CRM1 in carcinogenesis and review the new development of SINE inhibitiors in hematological malignancies.

### CRM1/exportin 1/XPO1

CRM1 was first identified in early 90s. A mutation in CRM1 gene was found to be affecting the chromosome structure in the yeast *schizosaccharomyces pombe*. Hence it was named as chromosome region maintenance [6]. However, its importance as an exportin was discovered later [7]. The discovery of Leptomycin B, a natural CRM1 inhibitor, aided the recognition of several CRM1 cargos [8]. Among all the exportins, CRM1 alone is responsible for transporting most of the tumor suppressors and growth regulators including p53, p21, FOXO, PI3K/AKT, Wnt/ß-catenin, AP-1 and NF-kB, etc.

The structural model of the human CRM1 was studied by X-ray crystallography modelling and electron microscopy [9]. CRM1 is a 120 kDa protein. It has 21 HEAT repeats. Each HEAT repeat consists of two antiparallel alpha helices termed A and B connected by an intraloop. CRM1 comprises of a ring like structure, in which the A helices form the outer convex and B helices the inner concave [10]. The outer convex surface contains a hydrophobic groove formed by HEAT repeats 11 and 12 that bind to NES peptides. What is crucial to the CRM1 structure is the presence of helices with diverse conformations such as the B helix of H21. For example, in the CRM1-cargo-RanGTP complex the H21B lies next to the H21A. However, this conformation is not in the CRM1-cargo complex without RanGTP [10].

Cargo proteins contain a leucine-rich nuclear export signal (LR-NES) that is recognized by CRM1. The LR-NES is a combined alpha-helical-extended peptide that resides in a hydrophobic groove between the two outer helices of CRM1 comprising of HEAT repeats 11 and 12 [1]. Now, more than 200 NES structures have been identified [2].

### Selective inhibitors of nuclear export (SINEs)

Leptomycin B (LMB) was the first CRM1 inhibitor identified. Other natural inhibitors include ratjadone C, anguinomycin, goniothalamin, along with the small molecule inhibitors, N-azolylacrylates and CBS9106. These inhibitors bind covalently to the cysteine residue (Cys528) in the NES-binding groove of the human XPO1. The binding prevents the loss of TSPs and stops cell proliferation [11,12]. However, LMB could not be considered as a therapeutic agent due to severe dose limiting toxicities like anorexia, nausea and vomiting [13].

**Figure 1:**
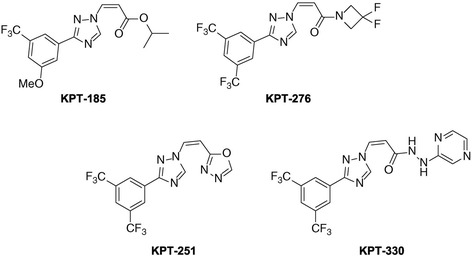
**The chemical structures of selective inhibitors of nuclear export (SINE).** Selinexor (KPT-330) is the first-in-human SINE agent in clinical trials. It is an oral small molecule inhibitor targeting CRM1/XPO1, the major nuclear-cytoplasmic exportin.

In recent years, novel small molecule SINE compounds that target CRM1 exportins have been developed, including KPT-185, KPT-251, KPT−276, KPT-330 (selinexor) and KPT-335 [3,14,15] (Figure 1). KPT-185 has been extensively investigated *in vitro*. However, *in vivo* study of this compound was limited by poor pharmacokinetics. This led to the development of KPT-330, the first SINE to reach clinical trials.

SINEs are now being explored in many clinical studies on both solid and hematologic malignancies. In this review, we discuss their potential as therapeutic agents in blood cancers.

#### SINEs in acute leukemias

The loss of tumor suppressors such as p53 has long been associated with cellular immortalization and proliferation. p53 promotes cell cycle arrest, apoptosis, autophagy and differentiation [16].

In recent years, various gene mutations in FLT3 (Fms-like tyrosine kinase 3), DNMT3A, NPM1 (nucleophosmin 1), and CEBPA (CCAAT enhancer-binding protein-α) have been found to play important roles in leukemognenesis [17-20]. NPM1 is a phosphoprotein that shuttles between the nucleus and cytoplasm. It regulates the p53-ARF pathway. The exon 12 mutation of the NPM1 gene has been implicated in leukemogenesis. The mutation leads to delocalization of NPM1 from the nucleolus to the cytoplasm (NPMc+) [21].

In 2012, the effects of KPT-185 and KPT−276 on AML cell lines and primary blasts were studied *in vitro* and *in vivo*. NPMc + blasts were found to be most responsive to SINEs (IC 50 = 100 nm). However, blasts with wild type NPM1 were also found to be SINE sensitive. This highlighted the role of other TSPs, especially p53 [22]. CRM1 inhibitors also induced blast differentiation. This was gauged by measuring expression of proteins like CEBPA that are involved in leukemogensis [20].

The prognostic significance of CRM1 was studied in 511 patients with newly diagnosed AML [23]. The viability of AML cell lines was assessed after the addition of KPT-185. It was found to induce apoptosis in p53 wild-type cells but only marginally in p53 deficient ones. It was also noted that patients with FLT3 or NPM1 mutations had higher levels of CRM1. High CRM1 was found to be an independent predictor of poor overall survival in AML patients. The study also commented on the synergistic approach of combining SINEs with Nutlin 3a, which is a MDM2 (mouse double minute 2) inhibitor [23].

SINEs were also studied in a panel of 14 human T-ALL cell lines, including Jurkat, and MOLT4 [24]. SINEs promoted cell cycle arrest in G1 phase and induced rapid apoptosis *in vitro*. KPT −330 was studied in mice bearing ALL or AML. KPT-330 demonstrated significant survival benefit in these mice [24].

The effects of KPT-330 on Philadelphia chromosome positive leukemia was studied in preclinical and clinical specimens [25]. CRM1/XPO1 expression was markedly increased in CML-BC, Ph(+) B-ALL as well as in Ph(−) B-ALL. CRM1/XPO1 expression was increased mostly in a TKI-sensitive manner in these cells. KPT-330 enhanced apoptosis and decreased the clonogenic potential of leukemic, but not normal, CD34(+) progenitors. The survival of BCR-ABL1(+) mice was found to be better with KPT-330 treatment. Half of the KPT-330 treated mice remained alive and, mostly, became BCR-ABL1 negative.

#### SINEs in chronic leukemias

The activities of SINEs were scrutinized in chronic lymphoid leukemia (CLL) in a preclinical study [12]. SINEs curbed cell growth by forcing the nuclear retention of major TSPs like p53, IkB and FOXO. KPT-185 resulted in the down regulation of MCL-1 expression in CLL cells. KPT-185 and KPT-251 were also examined in stromal cells such as HS-5 [12]. SINEs increased overall survival rate in the Emu-TCL1-SCID mouse model of CLL with minimal toxicities. Therefore, CRM1/XPO1 is a valid target in CLL with minimal effects on normal cells. This favors further development of SINEs in CLL and related hematologic malignancies [12].

KPT-330 (selinexor) was given to a 37 year-old male patient with TKI resistant CML-AP as a compassionate use since this patient has failed 9 prior therapies and declined bone marrow transplantation [25]. The patient received KPT-330 on a dose-escalation scale, but declined further treatment after a week. This represents one of the early experience in TKI-resistant CML patients.

#### SINEs in multiple myeloma (MM)

CRM1 is highly expressed and negatively correlates to survival in MM. In a preclinical study, CRM1 inhibition by SINE was explored in MM cells and in SCID mice. Higher levels of CRM1 were associated with bortezomib resistance, lytic bone disease and shorter survival. Blocking CRM1 activity by SINEs induced apoptosis in isolated MM cells and in those cultured in a simulated bone marrow microenvironment [26]. KPT-330 and KPT-185 were found to directly block osteoclastogenesis and bone resorption without adverse effects on osteoblastogenesis. This study also defined the role of CRM1 in osteoclast formation. Both KPT-185 and KPT-330 inhibited NF-ĸB and NFATc1, thus preventing the formation of a functional osteoclast [26]. This study also noted a synergistic effect when SINEs were combined with bortezomib [26].

KPT-276 is known to downregulate the various oncogenes, such as c-myc, cell division cycle 25 homolog A (CDC25A) and bromodomain-containing protein 4 (BRD4). KPT-276 significantly reduced the viability of 12 human myeloma cell lines [27]. KPT-276 also enhanced apoptosis in primary MM cells from patients. KPT-276 reduced monoclonal spikes and inhibited tumor growth in a xenograft MM mouse model. A phase I clinical trial of an analog of KPT-276 is ongoing in hematological malignancies including MM.

#### SINEs in lymphoma

CRM1 is overexpressed in various lymphomas as well. Lymphoma cell lines treated with SINEs had decreased viability, irrespective of p53 function. Oral administration of KPT-276 at 75 and 150 mg/kg resulted in significant tumor reduction [28]. Subcutaneous injections of KPT-251 (25 and 75 mg/kg) in mouse model resulted in significant suppression of lymphoma growth; residual tumors showed activation of the protein 73 pathway. This study provided evidence that CRM1 can serve as a therapeutic target in non-Hodgkin’s lymphoma (NHL) [28].

The effects of SINEs on mantle cell lymphoma (MCL) were also studied [29,30]. Compared with normal cells, MCL cells had higher XPO1 expression [29]. KPT-185 was found to enhance apoptosis of MCL cells through increased nuclear p53 levels [29]. Oral administration of KPT-276 significantly inhibited MCL growth in a mouse model without severe toxicity [30]. KPT-276 increased nuclear retention of CRM1.

A phase I trial of KPT-335 was done in 17 dogs with lymphoma, mast cell tumor or osteosarcoma. KPT-335 was administered at an oral dose of 1.75 mg/kg twice weekly. The common side effects of anorexia, weight loss, vomiting and diarrhea were manageable with supportive care [31].

KPT-330 (selinexor) was recently reported in a phase I trial in 32 patients with heavily pretreated NHL [32]. The median age of the patients was 68; median prior regimens: 3 (range 1–11). These patients received KPT-330 across 8 dose levels (3 to 60 mg/m^2^). Thrombocytopenia (20%) and neutropenia (20%) were the dose-limiting grade 3 and 4 adverse events. Gastrointestinal toxicities were the most common adverse events. Biomarker study from tumor biopsies confirmed nuclear localization of tumor suppressor protein, c-myc reduction, and apoptosis.

## Conclusion and future directions

Currently, selinexor (KPT-330) is undergoing several phase I and phase II clinical trials in both solid and haematological malignancies (clinicaltrials.gov). Selinexor represents a novel class of anti-cancer agents [3,14,15,33].
